# A Novel Approach to the Involvement of Loved Ones in the Treatment of Individuals with Eating Disorders: The Family Integration Model

**DOI:** 10.3390/bs16071049

**Published:** 2026-06-24

**Authors:** Renee D. Rienecke, Maria Bazo Perez, Amy Boyers, Wendy Oliver-Pyatt

**Affiliations:** 1 Galen Hope, Coral Gables, FL 33134, USA; mbazoperez@galenhope.com (M.B.P.); amy@galenhope.com (A.B.); wendy@galenhope.com (W.O.-P.); 2 Department of Psychiatry and Behavioral Sciences, Northwestern University, Chicago, IL 60611, USA

**Keywords:** eating disorders, support people, family, treatment outcomes, higher levels of care

## Abstract

The purpose of this study was to describe a novel approach to involving family members and support people in higher-level care treatment for patients with eating disorders (EDs) across the age spectrum, to examine outcomes on ED symptoms, depression, and anxiety, and to assess outcomes for patients presenting with high-severity symptoms. Family members also rated their satisfaction with family-specific programming. Participants were 137 adults and 61 adolescents receiving treatment at the intensive outpatient program and partial hospitalization program levels. Self-report measures of ED symptoms, depression, and anxiety were completed at admission and discharge. Adults and adolescents reported significant reductions on the Eating Disorder Examination-Questionnaire Global Score and all subscales from admission to discharge (all *p*s < 0.001). Adults (*t*(90) = 9.21, *p* < 0.001) and adolescents (*t*(36) = 4.27, *p* < 0.001) reported significant reductions in depression from admission to discharge (*p*s < 0.001). Adults (*p* < 0.001), but not adolescents (*p* = 0.07), reported significant reductions in anxiety. Overall, similar results were found for patients presenting with high-severity symptoms. Family members reported high levels of satisfaction with family programming. Findings suggest that this novel approach to involving family members and support people for those with EDs across the age spectrum may result in favorable treatment outcomes.

## 1. Introduction

The ego-syntonic nature of many eating disorders (EDs) (Halmi, 2013) can make it challenging for those struggling with these disorders to recover without support. As opposed to early conceptualizations of ED as being caused by dysfunctional families (Hoste et al., 2012), the field has moved decisively away from this viewpoint, now seeing family members as crucial to the recovery of those with EDs (Le Grange et al., 2010). The leading evidence-based treatment for children and adolescents with anorexia nervosa (AN), family-based treatment (FBT), temporarily puts parents in charge of the weight restoration process because the nature of the ED does not allow the affected individual to eat in a healthy and recovery-oriented manner (Lock & Le Grange, 2013). Parents in FBT thus play a central and crucial role in the treatment and recovery of their child.

Whitney and Eisler (2005) describe the profound impact that an ED has on family life, including the way in which the illness becomes the “central organizing principle” (p. 577) of the family members’ lives. As the ED progresses and caregivers attempt to cope, it is not unusual for caregivers—with good intentions—to start accommodating the disorder. They may drive out of their way to grocery shop for a specific low-fat yogurt that their child is willing to eat. They may allow their child to eat less than they should, because “at least they are eating something”. This behavior is understandable given the power of the ED, but it is not helpful. Accommodating behavior on the part of caregivers has been hypothesized to be a maintaining factor for AN in the cognitive-interpersonal maintenance model proposed by Schmidt and Treasure (2006) and has been supported by research showing that patient treatment outcomes are worse if parents are high on accommodation (Anderson et al., 2021; Salerno et al., 2016). FBT not only aids in the child gaining weight and/or stopping ED behaviors, it also addresses these accommodating behaviors on the part of the caregivers, educating caregivers about the crucial role that they play in treatment and showing them how to be more effective in their interactions with the ED.

Including support people in treatment for patients with EDs is important not only for the patient but also for the support person. Caregivers of those with EDs experience considerable psychological distress and caregiver burden (Zabala et al., 2009), helplessness and self-blame (Batchelor et al., 2022), often feel unsupported by the healthcare system, and experience uncertainty about whether they are managing the ED in the most effective way (Karlstad et al., 2021). They have a large number of unmet needs, primarily in the domains of practical and emotional help (Haigh & Treasure, 2003). A large study of Australian caregivers found that 96% of respondents reported worse emotional health than usual, 70.5% reported worse physical health than usual, 92.7% reported a deterioration in their romantic relationship, and 91.8% reported incurring significant out-of-pocket expenses for treatment (Wilksch, 2023). Caregivers, whether they are parents, other family members, friends, partners, or spouses, and regardless of whether they are supporting children, adolescents, or adults, have taken on a difficult role and are asking for training, information, and practical guidance in order to best support their loved one (Batchelor et al., 2022).

FBT has been studied in children, adolescents, and transition-age youth (Dimitropoulos et al., 2018). It is not designed for adults; however, adults with EDs also need support navigating their recovery journey. As with children and adolescents, the UK’s NICE guidelines (NICE, 2017) recommend the involvement of family members and/or carers in the treatment of adults with EDs. In recognition of this need, treatments have been developed to include spouses, family members, or support people of adults with EDs. Bulik et al. (2011), for example, created UCAN (Uniting Couples [in the treatment of] Anorexia Nervosa), which acknowledges that not only does the ED impact the patient’s partner, but the partner, wanting to help, may inadvertently do things that maintain or exacerbate the ED, similar to accommodating behaviors in caregivers of adolescents. Including the partner in treatment can help the couple improve communication and behaviors together. The Maudsley Model of Anorexia Nervosa Treatment for Adults (MANTRA) (Schmidt et al., 2014) and enhanced cognitive–behavioral therapy (CBT-E) (Fairburn & Beglin, 2008) also emphasize the importance of including support people during treatment for adults with EDs.

However, these are outpatient treatments. Involving caregivers and support people in the treatment of those with EDs receiving higher levels of care (HLOC) can be more challenging. For example, if a patient needs care at the inpatient, residential, or partial hospitalization level, they may need to travel to the treatment facility, or even attend treatment out-of-state, making the regular involvement of caregivers difficult. It is critical to involve them, however, so that they have the skills necessary to continue facilitating the recovery process once the patient returns home. The efficacy of FBT has led to the incorporation of FBT principles into HLOC programs (e.g., Easton et al., 2016; Girz et al., 2013; Hoste, 2015), but many of these programs are specifically for children and adolescents with EDs. There is a need for support people to be involved in the treatment of adults in HLOC programs as well.

The purpose of this paper is to describe a novel approach to involving family members and support people in HLOC treatment for patients with EDs across the age spectrum, to examine outcomes from admission to discharge for patients on ED symptoms, depression, and anxiety, and to assess outcomes for patients presenting with high-severity symptoms. It was hypothesized that both adults and adolescents would benefit from this novel treatment approach and would show significant improvements in ED, depression, and anxiety symptoms. It was also hypothesized that patients admitting above high-severity cutoffs on measures of ED, depression, and anxiety symptoms would show significant improvements over the course of treatment. In addition, quantitative satisfaction data from family members and support people involved in the treatment of their loved ones will be presented.

## 2. Materials and Methods

### 2.1. Participants and Procedure

Participants were 137 adults and 61 adolescents receiving treatment for an ED at a multisite treatment facility offering care at the intensive outpatient program (IOP) and partial hospitalization program (PHP) levels between October 2022 and January 2026. Self-report measures are completed at admission, monthly throughout treatment, and at discharge. Because measures are completed at monthly intervals, patients were eligible for inclusion in the study if they had completed at least 30 days of treatment and had a primary diagnosis of an ED. There were no other inclusion or exclusion criteria. This study was determined to be exempt by the Biomedical Research Alliance of New York Institutional Review Board (protocol # 25-166-2234).

### 2.2. Treatment

Patients are assigned to treatment at either the PHP or IOP level of care, depending on clinical and medical acuity and their percent expected body weight (%EBW). There are two PHP levels: one offers care 12 h per day, 7 days per week, and the other is 8 h per day, 5–7 days per week. The IOP consists of care for 4.5 h per day, 3–5 days a week. Treatment at the PHP levels includes weekly psychiatry visits, up to daily medical provider/nursing visits, twice weekly individual therapy sessions, weekly family therapy sessions, weekly sessions with a registered dietitian, 30 min of caregiver coaching weekly, 1–2 h of individual meetings with the patients’ assigned care partner, and 3–4 h of group therapy per day, including groups on dialectical behavior therapy (DBT), acceptance and commitment therapy (ACT), cognitive–behavioral therapy (CBT), and body image. Care partners are frontline staff who implement directives from the clinical, nutritional, and medical teams while providing structured, individualized support in daily routines, skill-building, and therapeutic follow-through (e.g., meals, exposures, and activities of daily living). Patients have three supervised meals and 3 supervised snacks per day, which include pre- and post-meal process groups and an emphasis on recognizing hunger/fullness cues. At the IOP level, patients receive 2–3 group therapy sessions daily, weekly individual appointments with a registered dietitian, and weekly sessions with their primary therapist. They are also followed by nursing to monitor weight and vitals. Patients have one snack and one meal per day while in programming.

### 2.3. Family Integration Model

In recognition of the importance of family members and support people in the recovery of patients with EDs, regardless of their age, the Family Integration Model takes a three-pronged approach to family involvement: (1) family therapy, (2) caregiver coaching, and (3) extensive psychoeducation exclusively for caregivers. These interventions occur in weekly family therapy sessions, biweekly family skills groups, biweekly caregiver support groups, and a monthly Family Day. Weekly family therapy is conducted with the patient and focuses on the patient’s struggles with the ED, psychoeducation about the function of the ED, communication skills, setting and holding boundaries, and learning DBT and CBT skills. The goal is for family members to leave treatment with a deep and thorough understanding of EDs and how their loved one is impacted by the disorder.

Biweekly family groups alternate between teaching caregivers DBT skills and offering support specifically for male loved ones of patients. The biweekly caregiver support groups aid caregivers in such areas as learning validation, accepting uncertainty, apologizing and forgiving, and understanding urge surfing. The monthly Family Day is a full day of interactive programming, including 3 h of psychoeducational programming, an experiential/supported lunch, a multifamily group, and a parent support/process group. The psychoeducational programming for caregivers covers a wide array of topics, including education about the roles of each care team member, information about ACT, DBT, and CBT, learning to understand anger, education about personality disorders, trauma symptoms, and treatment-undermining behaviors, and managing the transition from PHP down to IOP (see Appendix A). Most family members attend in person, even if they are coming in from out of town, although some are unable to attend in person and join virtually.

Caregiver coaching occurs once weekly for 30 min and occurs without the patient present. This is done to give the caregiver or support person private time and space to speak freely and process their feelings and experiences without running the risk of hurting their loved one. Coaching involves a practical piece, in which caregivers are given education about EDs, an instructional piece, where they are given skills to enable them to be more effective in their interactions with their loved one (for example, this may involve role playing), and an emotional processing piece, as caregivers are under considerable stress and need a space to process and address their stress. Extensive psychoeducation happens throughout treatment in the form of therapy, coaching, support/skills groups, and Family Day.

Parents or another adult caregiver are generally the support people for children and adolescents in the program, while adults may choose a spouse/partner or another supportive family member or friend. The Family Integration Model programming is the same for adolescent and adult patients. Active family participation is required, with the goal of supporting the wellbeing of every family member and allowing the family to heal together. Family members are fully integrated with the team and treatment program in a way that makes them feel valued and part of the recovery process.

### 2.4. Measures

The Eating Disorder Examination-Questionnaire (EDE-Q) (Fairburn & Beglin, 1994, 2008) is a widely used 28-item measure assessing the cognitive and behavioral psychopathology of EDs over the previous 28 days. Twenty-two items are scored on a scale of 0–6, with higher scores indicating more severe symptomatology. Six items assess the frequency of ED behaviors, such as binge eating or purging. The EDE-Q consists of four subscales: Restraint, Eating Concern, Shape Concern, and Weight Concern, and a Global Score.

The Patient Health Questionnaire-9 (PHQ-9) (Kroenke et al., 2001) is a 10-item self-report measure of depressive symptoms based on DSM-IV criteria for major depressive disorder (APA, 1994). Nine items are measured on a 4-point scale from 0 (“not at all”) to 3 (“nearly every day”), and a tenth item inquires about functional impairment due to depressive symptoms. Categories of severity include “minimal depression” (scores of 0–4), “mild depression” (scores of 5–9), “moderate depression” (scores of 10–14), “moderately severe depression” (scores of 15–19) and “severe depression” (scores of 20–27). In the current study, the PHQ-9 was given to patients aged 18 and older.

The Patient Health Questionnaire for Adolescents (PHQ-A) (Johnson et al., 2002) assesses depressive symptoms among adolescents. It is scored similarly to the PHQ-9, but the wording of certain items is somewhat different; for example, the first item, “feeling down, depressed, irritable, or hopeless?” includes irritability, whereas the PHQ-9 does not. In the current study, the PHQ-A was given to patients below the age of 18.

The Generalized Anxiety Disorder-7 (GAD-7) (Spitzer et al., 2006) is an 8-item self-report measure of generalized anxiety. Seven items are assessed on a 4-point scale from 0 (“not at all”) to 3 (“nearly every day”). The eighth item inquires into functional impairment as a result of anxiety. Categories of severity are “minimal anxiety” (scores of 0–4), “mild anxiety” (scores of 5–9), “moderate anxiety” (scores of 10–14), and “severe anxiety” (scores of 15–21).

### 2.5. Carer Satisfaction

Family members and support people are asked to rate their experience of the Family Integration Model Family Day on seven domains: (1) the staff psychoeducational groups, (2) the multifamily group, (3) the working lunch, (4) the family process group, (5) learning effective, healthy, and supportive communication skills, (6) communication with staff, and (7) Family Day overall. Ratings are made on a 5-point scale: 1 = very dissatisfied, 2 = dissatisfied, 3 = neutral, 4 = satisfied, and 5 = very satisfied. Information on carer satisfaction was collected between March 2023 and February 2026.

### 2.6. Statistical Analyses

To examine pre- to post-treatment change in self-reported ED, depressive, and anxiety symptoms, paired-samples *t*-tests were conducted comparing admission and discharge scores on the EDE-Q (Global and subscale scores), the PHQ-9 for adults and the PHQ-A for adolescents, and the GAD-7. The sample consisted of clients who completed 30 or more days of treatment and had a primary diagnosis of an ED, yielding a final sample of 198 clients (*n* = 61 adolescents; *n* = 137 adults). Analyses were conducted separately for adults and adolescents and for the sample overall. To account for multiple comparisons, the Bonferroni correction was applied within each family of tests, and corrected *p*-values were analyzed. A subgroup analysis was conducted, including clients whose admission EDE-Q Global Score exceeded the established clinical cutoff (>2.8; Mond et al., 2008; Velkoff et al., 2023), to evaluate whether treatment response differed for those presenting with more severe ED pathology at admission. Subgroup analyses were also conducted for clients scoring above the “severe” cutoff of 20 on the PHQ-9, above the “severe” cutoff of 20 on the PHQ-A, and above the “severe” cutoff of 15 on the GAD-7. Missing data were handled via pairwise deletion, so each paired *t*-test was conducted using all available cases with complete admission and discharge data for that specific measure, and therefore, analytic sample sizes varied by measure. Shapiro–Wilk tests indicated significant departures from normality across all measures and age groups. Given the moderate sample sizes and robustness of the paired *t*-test to non-normality, paired *t*-tests were retained as the primary analysis. Post hoc power analyses were conducted using the pwr package in R Studio v. 2025.05.1+513 (Champely, 2020), with Bonferroni-corrected alpha levels. Primary analyses demonstrated excellent statistical power across adult and adolescent samples. Specifically, all adult analyses (not divided by severity) achieved power = 1.00 (PHQ-9: *n* = 91, *d* = 0.97; GAD-7: *n* = 94, *d* = 0.75), as well as the adolescent EDE-Q analyses (*n* = 48, *d* = 0.71–0.96). Adult PHQ-9 moderate, moderately severe, and severe severity subgroups also achieved power ≥ 0.979. Adolescent PHQ-A (*n* = 37, *d* = 0.70, power = 0.970) and adolescent EDE-Q higher-severity subgroup (*n* = 27, *d* = 0.90–1.63, power = 0.964–1.00) similarly demonstrated adequate power.

There are limitations in power for the following subgroup analyses: the adolescent GAD-7 analysis (*n* = 43, *d* = 0.34, power = 0.459) and all adolescent GAD-7 analyses by severity subgroups were underpowered; thus, non-significant findings in these analyses should be interpreted with caution. Additionally, lower-severity subgroups in both age groups, particularly those with *n* < 15, reflect the distribution of symptom severity at admission and are reported as exploratory. Minimal and mild anxiety severity subgroups showed near-zero effect sizes consistent with floor effects rather than insufficient power. All statistical analyses were conducted using R Studio v. 2025.05.1+513 (Posit Team, 2025).

## 3. Results

Table 1 presents the demographic characteristics of the patients. Overall, the group consisted primarily of cisgender females (78.2%) and was primarily Non-Hispanic White (69.5%).

### 3.1. Eating Disorder Symptoms

Adults reported significant reductions on the EDE-Q Global Score and all subscales from admission to discharge (all *p_bonf_* < 0.001, see Table 2). Effect sizes were large across all subscales (*d*s = 0.69–0.91). Specifically, significant reductions were observed for the Global Score (*t*(107) = 9.35, *p_bonf_* < 0.001, *d* = 0.90, 95% CI [0.98, 1.51]), Restraint (*t*(107) = 7.51, *p_bonf_* < 0.001, *d* = 0.72, 95% CI [1.00, 1.71]), Eating Concern (*t*(107) = 9.50, *p_bonf_* < 0.001, *d* = 0.91, 95% CI [1.06, 1.62]), Shape Concern (*t*(107) = 8.13, *p_bonf_* < 0.001, *d* = 0.78, 95% CI [0.94, 1.54]), and Weight Concern (*t*(107) = 7.15, *p_bonf_* < 0.001, *d* = 0.69, 95% CI [0.79, 1.40]). Adolescents similarly reported significant reductions on the EDE-Q Global Score and all subscales from admission to discharge (all *p_bonf_* < 0.001; see Table 2), with large effect sizes across all subscales (*d*s = 0.71–0.96). Significant reductions were observed for the Global Score (*t*(47) = 6.49, *p_bonf_* < 0.001, *d* = 0.94, 95% CI [0.90, 1.70]), Restraint (*t*(47) = 5.56, *p_bonf_* < 0.001, *d* = 0.80, 95% CI [0.89, 1.91]), Eating Concern (*t*(47) = 6.63, *p_bonf_* < 0.001, *d* = 0.96, 95% CI [1.01, 1.89]), Shape Concern (*t*(47) = 4.90, *p_bonf_* < 0.001, *d* = 0.71, 95% CI [0.68, 1.64]), and Weight Concern (*t*(47) = 5.22, *p_bonf_* < 0.001, *d* = 0.75, 95% CI [0.74, 1.66]).

### 3.2. Depression

Adults (*t*(90) = 9.21, *p_bonf_* < 0.001, *d* = 0.97, 95% CI [5.63, 8.72]) and adolescents (*t*(36) = 4.27, *p_bonf_* < 0.001, *d* = 0.70, 95% CI [1.97, 5.54]) reported significant reductions in depression on the PHQ-9/PHQ-A from admission to discharge (see Table 2).

### 3.3. Anxiety

Adults reported significant reductions in anxiety symptoms on the GAD-7 from admission to discharge (*t*(93) = 7.30, *p_bonf_* < 0.001, *d* = 0.75, 95% CI [3.27, 5.71]). Adolescents did not report significant reductions in anxiety on the GAD-7 from admission to discharge (*t*(42) = 2.21, *p_bonf_* = 0.066, *d* = 0.34, 95% CI [0.15, 3.43]) (see Table 2). Given the limited statistical power for this analysis (power = 0.459), this non-significant finding should be interpreted with caution and considered exploratory rather than evidence of no treatment effect.

### 3.4. Severity Subgroup Analyses

Clients with high EDE-Q scores at admission (above the clinical cutoff of 2.8) experienced significant reductions across the EDE-Q Global Score and all subscales (all *p_bonf_* < 0.001; *d*s = 0.83–1.29, see Table 3). Adults scoring above the “severe” cutoff of 20 on the PHQ-9 reported significant reductions in depression from admission to discharge (*t*(25) = 8.63, *p_bonf_* < 0.001, *d* = 1.69, 95% CI [8.96, 14.58]). Adults scoring above the “severe” cutoff of 15 on the GAD-7 reported significant reductions in anxiety from admission to discharge (*t*(31) = 6.93, *p_bonf_* < 0.001, *d* = 1.23, 95% CI [5.62, 10.31]).

Adolescents scoring above the clinical cutoff of 2.8 on the EDE-Q experienced significant reductions on the EDE-Q Global Score and all subscales (all *p_bonf_* ≤ 0.002; *d*s = 0.90–1.63, see Table 3). Changes from admission to discharge for adolescents scoring above the “severe” cutoffs on the PHQ-A (*t*(5) = 2.55, *p_bonf_* = 0.26) and GAD-7 (*t*(11) = 1.89, *p_bonf_* = 0.34) were underpowered (power = 0.378 and power = 0.286, respectively) and not statistically significant; therefore, these analyses should be considered exploratory.

**Figure 1:**
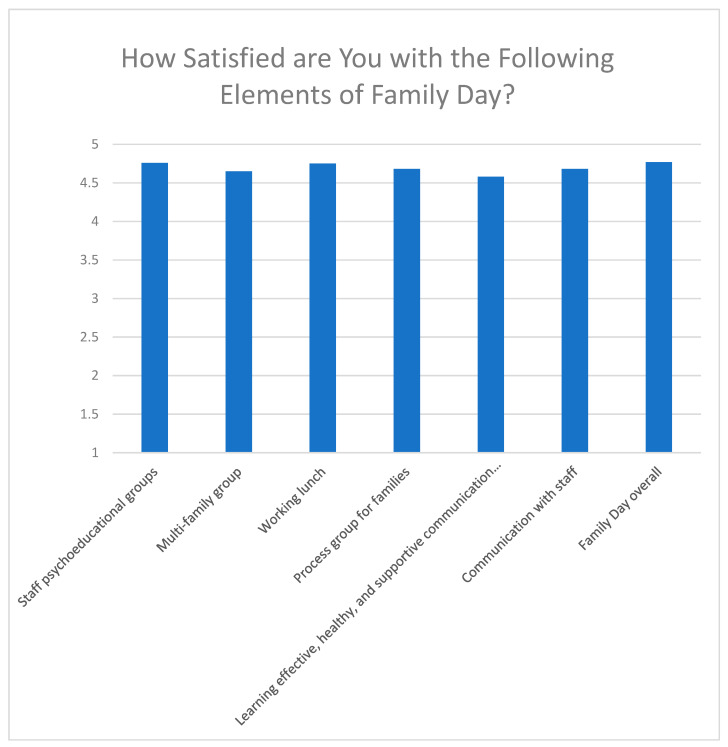
Carer Satisfaction with Family Day.

The Family Day carer satisfaction survey was completed by 174 family members and support people. Twenty-two Family Day participants did not complete the survey. Participants reported high levels of satisfaction with all aspects of Family Day (see Figure 1).

## 4. Discussion

This paper sought to describe a novel approach to the involvement of caregivers and support people in HLOC treatment for patients with EDs across the age spectrum. The hypothesis that both adolescents and adults would show improvement in ED symptoms, depression, and anxiety was largely supported. In addition, patients who were admitted to treatment with high-severity symptoms showed similar significant improvements.

Both the total sample of adults and those adults who presented with high-severity symptoms showed significant improvements on all measures after Bonferroni correction. Although family involvement has been recommended for adults receiving treatment for EDs (NICE, 2017), support people have generally been incorporated into outpatient therapies. It is more challenging in HLOCs to incorporate family support, often because patients may have to travel out of state for treatment. The results from this study suggest that this novel Family Integration Model may be helpful for both adults and adolescents struggling with EDs.

Adolescents, both the total sample and those presenting with high-severity symptoms, showed similar improvements on the EDE-Q, but less clear improvement on the measures of depression and anxiety. These findings are consistent with other outcome studies of adolescents with EDs receiving treatment in HLOC that show significant improvements in ED symptoms but less improvement on measures of depression and anxiety (Hoste, 2015; Reilly et al., 2024).

Findings that adult and adolescent patients presenting with high-severity symptoms on the EDE-Q showed significant improvements on this measure from admission to discharge are particularly encouraging, suggesting that improvements at this treatment program are not only being seen among those presenting with low- or moderate-severity symptoms. Similar to the findings from the overall groups of adults and adolescents, adult patients presenting above “severe” cutoffs on the PHQ-9 and GAD-7 improved on these measures, while adolescents presenting above these cutoffs did not.

It may be expected that patients with EDs would improve less on measures of depression and anxiety in a program that focuses primarily on reducing ED symptoms. In addition, feelings of distress and fear can occur during the recovery process for those with EDs (Christian et al., 2024), as patients face feared foods, eat regular meals, and, for those who need to gain weight, experience weight restoration. Although both adolescents and adults experienced significant improvements on all EDE-Q subscales, the scores on the EDE-Q Shape Concern and Weight Concern subscales are higher at discharge than the Restraint and Eating Concern subscales. This is also consistent with previous literature on patients with EDs in HLOCs (e.g., Hoste, 2015). The Restraint and Eating Concern subscales consist of many behavioral items (e.g., “Have you been deliberately trying to limit the amount of food you eat to influence your shape or weight (whether or not you have succeeded)?”) (underline in original). Restricting one’s food is much more difficult to do while in HLOC treatment. However, items on the Shape Concern and Weight Concern subscales tend to be more cognitive in nature (e.g., “Have you had a definite desire to have a totally flat stomach?”) (underline in original). These body image concerns may be challenged during ED treatment, which involves developing a regular pattern of eating and perhaps restoring weight. These subscales do improve with treatment, but they tend to remain higher than the other two subscales, and it would perhaps not be surprising if, near the beginning of treatment, they actually worsen before improving.

Although adolescents did not improve as much on depression and anxiety, the group of adults did make significant progress on these measures. This may be due to the fact that adults entering treatment are generally there of their own free will, as they must willingly enter treatment unless they are ill enough to be forcibly sent to treatment; this treatment center did not accommodate individuals with that level of illness. Adolescents, on the other hand, are often in treatment because their parents decided that they needed it. Adolescents report greater feelings of being coerced into treatment than do adults (Guarda et al., 2007), and older age has been found to be positively associated with desire for treatment among those with EDs (Casasnovas et al., 2007). Findings regarding the influence of depression on treatment outcomes for individuals with EDs are mixed. Depression has been found to predict poor treatment outcome at discharge and 1-year follow-up for adolescents and adults receiving residential or partial hospitalization treatment for an ED (Fewell et al., 2017), while depression was not found to predict treatment outcome for adults receiving inpatient treatment for AN (Calugi et al., 2014). In terms of anxiety, while anxiety disorders and EDs are frequently comorbid (Swinbourne & Touyz, 2007), there seems to be less evidence that anxiety may negatively impact treatment outcome for those with EDs. There are currently no clear guidelines for how best to manage co-occurring disorders in individuals with EDs: whether to focus primarily on the ED, to offer sequential interventions before or after treatment of the ED, or to integrate multiple interventions concurrently (Wade et al., 2024). Until research is conducted to determine the best way to treat multiple comorbidities for those with EDs, in order to improve outcomes for adolescents with depression and/or anxiety, it may be useful to develop separate tracks for those identified at intake as struggling with these comorbid symptoms, to develop additional groups to specifically address these symptoms, or to structure individual therapy sessions to incorporate evidence-based treatments for comorbid mood and anxiety issues in addition to the ED. Determining whether depression and anxiety predated the ED or seem to be a consequence of the ED could help to inform these treatment decisions.

Carers were highly satisfied with the Family Day programming, with one commenting, “(The treatment facility) goes to extraordinary lengths to engage client families at every step of the process.” Over 20 years ago, Dare and Eisler (2000) described the development of a multifamily group incorporated into the day treatment program for adolescents with EDs at the Maudsley Hospital in London. The principles of this multifamily group are similar to family-focused treatments for EDs and are designed to improve outcomes by reducing the isolation and perceived stigma often experienced by carers of those with EDs (Donkin et al., 2026; Highet et al., 2005) and promoting family skill building (Baudinet et al., 2021). A qualitative study of the experiences of patients with severe EDs and their families in multifamily group therapy identified two themes: “connectedness and recognition” and “opening up and sharing” (Brinchmann & Krvavac, 2021). A mother spoke to the power of the group: “The first time…we sat in our group…I began to cry…I think that it was because I realised that at last there were people who understood what I was talking about.” (Brinchmann & Krvavac, 2021, p. 6). Similarly, one Family Day participant wrote in the comments section of the survey: “Very informative and nice to have support from the other families.” Empirical studies also show evidence of the efficacy of multifamily therapy groups in the treatment of individuals with EDs (Baudinet et al., 2021; Eisler et al., 2016).

It has been well-established that carers and support people of those with EDs experience high levels of burden and distress (Wilksch, 2023; Zabala et al., 2009). Although the ED field has decisively moved away from blaming carers for causing EDs in their children, anecdotally, too many carers are still blamed by misinformed healthcare providers and excluded from their child’s treatment. The model described in this paper recognizes not only the support needed by carers of those with EDs but also the skills that they can bring to the treatment of their loved one. In addition, the Family Integration Model acknowledges the assistance to carers that can be provided by coming together with other families, and the relief that can come from knowing that they are not alone in their fight.

Limitations of the current study include the uncontrolled design and lack of a control group. Further, without a dismantling study, it is difficult to say to what degree the Family Integration Model is responsible for bringing about improvement above and beyond other evidence-based elements of the treatment program, such as CBT, DBT, or ACT. Due to the parameters of the clinical setting and patients paying for treatment with insurance, randomized controlled trials in which some patients have family involvement and others do not are not feasible in this treatment setting. This would be an important area for future research in settings that can accommodate this study design. Another important area for future research would be to assess the specific therapeutic aspects that the Family Integration Model is designed to help with, such as a greater understanding of EDs on the part of family members and support people. Strengths include the use of well-validated measures and the description of a novel way of integrating carers into a HLOC setting for both adolescents and adults.

## 5. Conclusions

The Family Integration Model described in this paper represents a powerful way of incorporating family members and support people into the treatment of individuals with EDs across the age spectrum, with promising outcomes for ED symptoms, depression, and anxiety. Patients presenting with high-severity symptoms showed significant improvements as well. This model recognizes not only the unmet needs and stress that carers often experience while their loved one is ill but also the desire that carers have to support and help their loved one through the recovery process. The Family Integration Model may serve as an example for other programs of how carers can be successfully incorporated into HLOC treatment for patients with EDs.

## Figures and Tables

**Table 1:** Demographic and Clinical Characteristics.

	Adolescents (*N* = 61)	Adults (*N* = 136)	Overall (*N* = 197)
Age (*M*, *SD*)	16.21 (2.09)	25.71 (8.09)	22.77 (8.1)
Length of Stay in Days (*M*, *SD*)	101.2 (75.26)	112.35 (102.22)	108.93 (94.73)
**Gender (*N*, %)**			
Cisgender Female	47 (77.0%)	107 (78.7%)	154 (78.2%)
Cisgender Male	10 (16.4%)	18 (13.2%)	28 (14.2%)
Transgender Female	0 (0.0%)	0 (0.0%)	0 (0.0%)
Transgender Male	0 (0.0%)	3 (2.2%)	3 (1.5%)
Nonbinary/Non-gender conforming/Agender	4 (6.6%)	8 (5.9%)	12 (6.1%)
**Race (*N*, %)**			
Non-Hispanic White	32 (52.5%)	105 (77.2%)	137 (69.5%)
White Hispanic/Latinx	23 (37.7%)	25 (18.4%)	48 (24.4%)
African American	3 (4.9%)	1 (0.7%)	4 (2.0%)
American Indian/Native American/Alaska Native	0 (0.0%)	0 (0.0%)	0 (0.0%)
Asian/Asian American	1 (1.6%)	2 (1.5%)	3 (1.5%)
Middle Eastern/Northern African	1 (1.6%)	1 (0.7%)	2 (1.0%)
Native Hawaiian/Other Pacific Islander	0 (0.0%)	0 (0.0%)	0 (0.0%)
Multiracial	1 (1.6%)	2 (1.5%)	3 (1.5%)
Unsure/Do not know/Prefer not to say	0 (0.0%)	0 (0.0%)	0 (0.0%)
**Diagnosis (*N*, %)**			
AN	39 (63.9%)	79 (58.1%)	118 (59.9%)
BN	9 (14.8%)	9 (6.6%)	18 (9.1%)
BED	2 (3.3%)	20 (14.7%)	22 (11.1%)
ARFID	3 (4.9%)	8 (5.9%)	11 (5.6%)
OSFED	5 (8.2%)	12 (8.8%)	17 (8.6%)
UFED	3 (4.9%)	6 (4.4%)	9 (4.6%)
Missing/NA	0 (0.0%)	2 (1.4%)	2 (1.0%)
**Admission Level of Care (*N*, %)**			
IOP	13 (21.3%)	5 (3.7%)	18 (9.1%)
PHP-8 h	18 (29.5%)	37 (27.2%)	55 (28.0%)
PHP-12 h	30 (49.2%)	91 (66.9%)	121 (61.4%)
Missing/NA	0 (0.0%)	3 (2.2%)	3 (1.5%)

Note. AN = anorexia nervosa; BN = bulimia nervosa; BED = binge eating disorder; ARFID = avoidant/restrictive food intake disorder; OSFED = other specified feeding or eating disorder; UFED = unspecified eating disorder; IOP = intensive outpatient program; PHP = partial hospitalization program. The total number of patients is 197 because demographic information was missing for one adult.

**Table 2:** Clinical Scores at Admission and Discharge.

	Adolescents				Adults			
	Admission *M* (*SD*)	Discharge *M* (*SD*)	*t*-Value, *p_bonf_*	Cohen’s *d* [95% CI]	Admission *M* (*SD*)	Discharge *M* (*SD*)	*t*-Value, *p_bonf_*	Cohen’s *d* [95% CI]
EDE-Q Global Score	2.93 (1.79)	1.63 (1.45)	6.49, <0.001	0.94 [0.90, 1.70]	2.96 (1.56)	1.72 (1.25)	9.35, <0.001	0.98 [0.98, 1.51]
EDE-Q Restraint	2.29 (1.96)	0.89 (1.27)	5.56, <0.001	0.80 [0.89, 1.91]	2.22 (2.01)	0.86 (1.34)	7.51, <0.001	0.72 [1.00, 1.71]
EDE-Q Eating Concern	2.45 (1.73)	1.00 (1.31)	6.63, <0.001	0.96 [1.01, 1.89]	2.45 (1.53)	1.11 (1.35)	9.50, <0.001	0.91 [1.06, 1.62]
EDE-Q Shape Concern	3.73 (1.90)	2.57 (1.99)	4.90, <0.001	0.71 [0.68, 1.64]	3.85 (1.74)	2.61 (2.01)	8.13, <0.001	0.78 [0.94, 1.54]
EDE-Q Weight Concern	3.25 (1.96)	2.05 (1.82)	5.22, <0.001	0.75 [0.74, 1.66]	3.35 (1.80)	2.25 (1.91)	7.15, <0.001	0.69 [0.79, 1.40]
PHQ-A/PHQ-9	10.38 (7.97)	6.62 (6.93)	4.27, <0.001	0.70 [1.97, 5.54]	14.41 (7.30)	7.23 (6.61)	9.21, <0.001	0.97 [5.63, 8.72]
GAD-7	9.26 (6.34)	7.47 (6.78)	2.21, 0.07	0.34 [0.15, 3.43]	11.66 (6.08)	7.17 (5.71)	7.30, <0.001	0.75 [3.27, 5.71]

Note. *N*s vary for each outcome due to missing data on some measures.

**Table 3:** Clinical Scores at Admission and Discharge for Patients Presenting with High-Severity Symptoms.

			Adolescents			Adults		
	Admission *M* (*SD*)	Discharge *M* (*SD*)	*t*-Value, *p_bonf_*	Cohen’s *d*, [95% CI]	Admission *M* (*SD*)	Discharge *M* (*SD*)	*t*-Value, *p_bonf_*	Cohen’s *d*, [95% CI]
EDE-Q Global Score	4.33(0.84)	2.32(1.52)	7.48, <0.001	1.44 [1.46, 2.56]	4.18(0.81)	2.41(1.51)	9.21, <0.001	1.20 [1.39, 2.16]
EDE-Q Restraint	3.60(1.55)	1.34(1.50)	6.42, <0.001	1.24 [1.53, 2.98]	3.47(1.72)	1.33(1.56)	8.48, <0.001	1.10 [1.64, 2.65]
EDE-Q Eating Concern	3.79(0.94)	1.46(1.53)	8.49, <0.001	1.63 [1.76, 2.88]	3.49(1.12)	1.54(1.47)	9.88, <0.001	1.29 [1.56, 2.35]
EDE-Q Shape Concern	5.15(0.79)	3.51(1.95)	4.68, <0.001	0.90 [0.92, 2.36]	5.09(0.85)	3.58(1.86)	6.39, <0.001	0.83 [1.04, 1.98]
EDE-Q Weight Concern	4.77(0.91)	2.96(1.86)	5.42, <0.001	1.04 [1.12, 2.49]	4.69(1.03)	3.31(1.87)	6.74, <0.001	0.88 [1.09, 2.02]
PHQ-A/PHQ-9	23.33(2.80)	15.67(8.80)	2.55, 0.257	1.04 [−0.06, 15.40]	22.73(2.13)	10.96(6.97)	8.63, <0.001	1.69 [8.96, 14.58]
GAD-7	17.67(2.15)	14.17(6.49)	1.89, 0.342	0.55 [−0.58, 7.58]	18.44(1.87)	10.47(6.15)	6.93, <0.001	1.23 [5.62, 10.31]

Note. *N*s vary for each outcome due to missing data on some measures.

## Data Availability

The data presented in this article are not readily available due to privacy reasons. Requests to access the data should be directed to Wendy Oliver-Pyatt.

## Appendix A

Caregiver Support Group Topics

- Accepting Uncertainty
- Anger
- Anxiety
- Apologizing and Forgiveness
- Attachment Styles
- Behavior Chaining
- Boundaries
- Communication Skills
- Conflict
- Core Beliefs
- Dialectical Behavior Therapy Skills
- Dialectics
- Harmful Behaviors/Antidotes
- Justifying/Arguing/Denying/Explaining
- Stages of Change
- Stages of Development
- Support
- Thinking Errors
- Urge Surfing
- Validation

Family Day Topics

- Acceptance and Commitment Therapy
- Adolescent Development
- Boundaries
- Cognitive–Behavioral Therapy
- Communication
- Dangers of Rushing Treatment
- Dialectical Behavior Therapy
- Movement
- Nutrition
- Personality Disorders
- Role of the Care Partner
- Role of the Care Team
- Sick Role
- Stepping Down to Intensive Outpatient Programming
- Trauma
- Treatment-Undermining Behaviors
- Understanding Anger
- Understanding Treatment
